# Implementing a digital health model of care in Australian youth mental health services: protocol for impact evaluation

**DOI:** 10.1186/s12913-021-06394-4

**Published:** 2021-05-12

**Authors:** Sarah Piper, Tracey A. Davenport, Haley LaMonica, Antonia Ottavio, Frank Iorfino, Vanessa Wan Sze Cheng, Shane Cross, Grace Yeeun Lee, Elizabeth Scott, Ian B. Hickie

**Affiliations:** The University of Sydney, Brain and Mind Centre, 94 Mallett St, Camperdown, Sydney, NSW 2050 Australia

**Keywords:** Mental health, Digital health, Digital health solution, Health information technology, Mental health services, Young people, Implementation, eHealth

## Abstract

**Background:**

The World Economic Forum has recently highlighted substantial problems in mental health service provision and called for the rapid deployment of smarter, digitally-enhanced health services as a means to facilitate effective care coordination and address issues of demand. In mental health, the biggest enabler of digital solutions is the implementation of an effective model of care that is facilitated by integrated health information technologies (HITs); the latter ensuring the solution is easily accessible, scalable and sustainable. The University of Sydney’s Brain and Mind Centre (BMC) has developed an innovative digital health solution – delivered through the Youth Mental Health and Technology Program – which incorporates two components: 1) a highly personalised and measurement-based (data-driven) model of youth mental health care; and 2) an industrial grade HIT registered on the Australian Register of Therapeutic Goods. This paper describes a research protocol to evaluate the impact of implementing the BMC’s digital health solution into youth mental health services (i.e. *headspace -* a highly accessible, youth-friendly integrated service that responds to the mental health, physical health, alcohol or other substance use, and vocational concerns of young people aged 12 to 25 years) within urban and regional areas of Australia.

**Methods:**

The digital health solution will be implemented into participating *headspace* centres using a naturalistic research design. Quantitative and qualitative data will be collected from *headspace* health professionals, service managers and administrators, as well as from lead agency and local Primary Health Network (PHN) staff, via service audits, Implementation Officer logs, online surveys, and semi-structured interviews, at baseline and then three-monthly intervals over the course of 12 months.

**Discussion:**

At the time of publication, six *headspace* centres had been recruited to this study and had commenced implementation and impact evaluation. The first results are expected to be submitted for publication in 2021. This study will focus on the impact of implementing a digital health solution at both a service and staff level, and will evaluate digital readiness of service and staff adoption; quality, usability and acceptability of the solution by staff; staff self-reported clinical competency; overall impact on *headspace* centres as well as their lead agencies and local PHNs; and social return on investment.

**Supplementary Information:**

The online version contains supplementary material available at 10.1186/s12913-021-06394-4.

## Background

Young people aged 12 to 25 years have the highest incidence and prevalence of mental illness across the lifespan and bear a disproportionate share of the burden of disease associated with mental disorders [1–3]. Most recently, the Australian Child and Adolescent Survey of Mental Health and Wellbeing (conducted in 2013–14) estimates that 560,000 children and adolescents aged 4 to 17 years (almost 14%) in Australia experienced a mental disorder in the 12 months before the survey [4]. Considering that 75% of serious mental illness, alcohol or other substance misuse occurs before the age of 25 years [5], early identification and intervention is crucial to prevent illness progression and reduce its impact. Left untreated, the ramifications of poor mental health can last a lifetime, often robbing the individual of their quality of life and costing the economy billions.

In response to this, the Australian Government established the National Youth Mental Health Foundation (*headspace*) in 2006 – with an aim to establish a highly accessible, youth-friendly, multi-disciplinary service that responds to the mental health, physical health, alcohol or other substance use, and vocational concerns of young people aged 12 to 25 years [6]. *headspace* aims to better integrate and coordinate the appropriate care for a young person, and ensure early detection and intervention of emerging mental and substance use disorders [6]. To achieve this, centres are staffed by multidisciplinary teams to address the multidimensional needs of young people, comprising of clinical staff (e.g. psychiatrists, mental health nurses, GPs; allied health professionals such a psychologists, occupational therapists, social workers), and non-clinical staff (e.g. centre manager, clinical coordinator, community outreach worker, intake workers, etc) [7]a. The composition and capacity of these teams vary across *headspace* centres nationally, due to factors such as geographical location (e.g. unavailable workforce in rural locations), funding arrangements, and the fact that the provision of treatment and services offered by a *headspace* centre is determined by the community in which it is located – i.e., *headspace* centres aim to reflect and serve the communities in which they are based. Despite the establishment of *headspace*, mental health outcomes for young people remain poor. For those who access care, a significant portion of young people receive an inappropriately low level of treatment for their needs [8], show no improvement in functioning over time [9], or deteriorate to a more serious stage of illness [10, 11].

Poor outcomes for young people with mental ill health can occur for a variety of reasons, many of which stem from the current mental health system’s reliance on a ‘traditional’ model of service delivery (i.e. accessed in a ‘bricks and mortar’ service, utilising a waitlist, delivering exclusively face-to-face therapy, etc.) [12]. Within these traditional models of service delivery, not all Australians have access to high quality mental health care, and access to care varies greatly across geographical locations [13]. For example, people living in non-urban (regional, rural, remote) areas of Australia have limited access to mental health care [14] due to barriers such as location of service, service opening hours, the issue of anonymity (particularly in small rural or remote communities), stigmatising attitudes, and cost [15–17], and they experience higher self-harm, suicidal ideation, and suicide attempt rates than Australians living in urban areas [4]. Further, the quality and type of intervention received by a young person varies greatly across services and geographical locations [13]. There is limited communication and coordination between services, which leaves the young person navigating a disjointed mental health care system without support [18].

Recently, the World Economic Forum highlighted the substantial problems in mental health service provision and called for the rapid deployment of smarter, digitally-enhanced mental health services as a means to facilitate effective care coordination and address issues of demand [19]. A rapid increase in reported mental health issues during the COVID-19 pandemic has added to service demand, which cannot be met by current traditional service delivery models of mental health care [19]. Moreover, this surge in service demand has resulted in a significant increase in contacts with digital mental health services (e.g. Mindspot, Lifeline), further highlighting the important role digital health can play in providing mental health care on a large scale [20]. Yet, this increase also illustrates the need for better models of care otherwise we risk bringing more people into an already over-burdened mental health system. Thus, the adoption of a digital health model of care by mental health services, and a shift in the historically cautious attitudes of clinicians toward the use of digital technologies [19], is now crucial in facilitating quality, timely, and easily accessible mental health care for all.

### Youth mental health and technology program

The integration of health information technologies (HITs) into service delivery pathways is the biggest enabler of digital health [21–23], due to HITs being easily accessible, scalable, and sustainable. HITs have been defined as ‘the application of information processing involving both computer hardware and software that deals with the storage, retrieval, sharing, and use of health care information, data and knowledge for communication and decision making’ [24]. HITs can be utilised in a variety of health care settings (e.g. private and public sectors, primary, secondary and hospital services), and examples of HITs include electronic health records, medical billing software, and clinical decision support (a data-analysis system designed to support health professionals to make clinical decisions). Previous research has identified the key uses of HITs as being the storage, management, and transmission of health data; clinical decision making support; and the facilitation of health care from a distance [25]. HITs are currently transforming both the healthcare system and the ability of consumers to self-manage aspects of their health and wellbeing through exercising greater choice and control in their own care [26–30]. For mental health service providers and health professionals who employ shared decision-making, HITs are also transforming the ways in which consumers can choose to become active participants and equal partners in their health care, shifting service delivery from an intervention model to an effective coordinated care model that is person-centred and quality-driven [29–32]. The adoption of HITs in youth mental health services has already demonstrated their utility to improve access and triage to care, as well as communication between health professionals and consumers, particularly in regional, rural or remote settings [17].

Researchers at The University of Sydney’s Brain and Mind Centre (BMC) have developed the Youth Mental Health (YMH) and Technology Program which utilises an innovative digital health model of care to increase access to quality mental health care for young people, improve mental, physical and social outcomes for young people, and upskill the youth mental health workforce to confidently deliver quality care. This digital health solution – delivered through the YMH and Technology Program – incorporates two components. The first is our highly personalised and measurement-based (data-driven) model of youth mental health care (known as the *BMC Youth Model*) [33], which has been developed from more than 10 years of clinical research from the BMC’s Optymise Youth Cohort [11, 34]. The *BMC Youth Model* integrates a variety of clinical concepts, including a multidimensional assessment and outcomes framework (i.e. fostering holistic practice of mental health that considers other compounding comorbidities such as physical health), underlying pathophysiological mechanisms and illness trajectories (i.e. adopting a transdiagnostic framework that recognises that mental health conditions are rarely homogeneous), and clinical staging for mental health. Clinical staging models consider the spectrum of mental ill health and aim to place consumers on that continuum, from those with risk factors and symptoms or impairment (Stage 1a) or attenuated disorders (Stage 1b) through to those with discrete disorders or with persistent and recurrent syndromes (Stage 2+) [34]. Clinical stage separates young people based on differential risk of progression to more severe disorders and poorer outcomes, and is therefore an accurate and efficient guide to allocating care (i.e. a concept known as ‘staged care’) [35]. As a result of this research, the *BMC Youth Model* was subsequently translated into an education and training program [36], and its core concepts developed as key functionalities within an innovative HIT known as the InnoWell Platform [23, 37].

The second component of the digital health solution is a HIT, as exemplified by the InnoWell Platform [23, 37]. The InnoWell Platform is listed on the Australian Register of Therapeutic Goods (software as a medical device, class 1, ARTG ID 315030) as a customisable digital toolkit to assist assessment, monitoring, and management of mental ill health and maintenance of wellbeing. It does this by collecting, storing, scoring, and reporting personal and health information back to consumers and their health professionals to promote collaborative care partnerships [38]. The clinical content is determined by service providers. Importantly, the InnoWell Platform does not itself provide stand-alone medical or health advice, diagnosis, or treatment. Instead, it guides and supports, but does not direct, consumers and their treating health professionals to decide what may be suitable care options [38]. The InnoWell Platform is manufactured by InnoWell Pty Ltd. – a joint venture between the University of Sydney and PwC (Australia).

Therefore, our digital health solution facilitates use of the *BMC Youth Model* through the InnoWell Platform. Though we reference the InnoWell Platform as an exemplar HIT, it is important to note that the *BMC Youth Model* can be adopted via any HIT so long as its design has been guided by similar clinical and scientific concepts to provide highly personalised and measurement-based care.

This study aims to evaluate the impact of implementing the digital health solution (delivered through the YMH and Technology Program) into participating *headspace* centres within urban and regional areas of Australia. It focuses on impact at both a service and staff level, and will evaluate: digital readiness of service and staff adoption; quality, usability and acceptability of the solution; staff self-reported clinical competency; overall impact on *headspace* centres, their lead agencies and local Primary Health Networks (PHNs); as well as social return on investment.

## Methods

### Study design and procedure

#### Implementation of the digital health solution

The Implementation of the digital health solution into participating centres will be guided by our previously developed strategy for implementation science [39]. This strategy was developed and tested through previous research that implemented a HIT across a range of services and associated populations, including youth mental health (*headspace*), the veterans community (Open Arms – Veterans and Families Counselling), and eating disorder support services (Butterfly’s National Helpline), as part of Project Synergy (a three year, Australian government -funded project which aimed to transform mental health services through the use of technology [23, 38]).

The implementation strategy consists of four phases: Phase 1) scoping and feasibility (determine the fit regarding the aims of the service and the solution provided by the HIT); Phase 2) utilise co-design methodologies (including participatory design, service pathway modelling and user testing) to determine how the HIT can enhance the service; Phase 3) implement the HIT into the service, iteratively evaluating the solution over time to respond to the needs of the service (including staff, individuals, and individuals’ support people); and Phase 4) the sustainment of the optimised HIT within the service (informed by phases 1 to 3), to achieve mental health service transformation through technology [39].

Implementation will follow a naturalistic research design, with each participating centre utilising the digital health solution for a period of 12 months. The naturalistic design will allow the digital health solution to be evaluated under ecologically valid conditions that reflect the customisable and changeable nature of the digital health solution in a ‘real-world’ youth mental health service setting. The order in which centres will implement the digital health solution will be naturalistically guided by each centre’s unique circumstance, as per Phase one of our implementation strategy, ‘scoping and feasibility’. Participating services will be offered the option of continuing to utilise the digital health solution as part of service delivery after the 12 month implementation period, without the accompanying impact evaluation research measures.

#### Participating centres

A minimum of six *headspace* centres across Australia will be offered the opportunity to implement the digital health solution into their service. Centres will be selected based on interest from the centres’ commissioning body (their Primary Health Network [PHN]). The study’s Coordinating Principal Investigator, supported by the study’s co-investigators, will approach the *headspace* centre’s relevant contact (e.g. service manager), via phone or email to offer further information regarding the implementation of the digital health solution. Centre managers will be contacted directly to ensure no selection bias with regards to PHN involvement. Centres will then commence phase one of the implementation science strategy (scoping and feasibility) [39].

#### Impact evaluation

Impact evaluation of the digital health solution has been guided and adapted by our previous work [40], which established data collection methods specifically to evaluate the impact of HITs for Australian mental health services reform. Methods include service audits, Implementation Officer logs, online surveys, and semi-structured interviews, allowing us to collect longitudinal quantitative and qualitative data. This unique use of methodological triangulation (i.e. mixed methods) ensures that data collection will be comprehensive and inclusive of all stakeholders to drive enhanced understanding of the potential impacts of implementation. The triangulation of data has previously been used to evaluate the implementation of HITs amongst health service professionals [41, 42].

##### Service audits

Service audits will collect non-identifiable and aggregated data from participating *headspace* centres’ relevant electronic health records software (e.g. Best Practice, Medical Director) to assess domains of clinical safety and service quality, such as accessibility and equity, acceptability and satisfaction, workforce competence and capability, and so on.

##### Implementation officer logs

Implementation Officers, part of the Youth Mental Health and Technology research team at the University of Sydney’s Brain and Mind Centre, will be embedded into participating *headspace* centres to support implementation. Implementation Officers will have a background working in the provision of mental health services (either clinical or non-clinical [e.g. administrative, research-focussed]), and will be embedded into participating centres part-time, contingent upon the varying needs and capacities of the services. Other tasks of Implementation Officers will include acting as a point of contact for participating sites, managing the recruitment of participants, overseeing informed consent, distributing online surveys to participants, administering semi-structured interviews, and routinely completing the Implementation Officer Log. The Implementation Officer log is based on the Quality Implementation Framework [43], and will be used to monitor changes in implementation, reimagined service pathways, and staff roles related to the digital health solution. Importantly, Implementation Officers are not making any observations about centre staff specifically (e.g. performance), but rather the processes of implementing the digital health solution within participating *headspace* centres. Implementation Officers will complete logs fortnightly through REDCap, a secure web application for building and managing online surveys and databases [44, 45]. Table 1 provides an example of the range of questions included in the Implementation Officer log, a full version of the log is provided in Additional file 1: Appendix A.

**Table 1:** Example questions from the Implementation Officer log

*All questions are asked regarding the Implementation Officer’s experience over the past two weeks.*	
Category	Question
Service-level impacts	Is the digital health solution changing and/or improving the following aspects of the mental health service? If yes, how? If no, why not? i) Clinical safety ii) Accessibility iii) Continuity of care iv) The delivery of staged care v) Etc.
Capacity/readiness	Have there been any changes to the service’s capacity (e.g. resources, skills, motivation)? What changes?
Quality and usability of the digital health solution	Does the platform require modifications to improve its performance? Yes/No, describe. Does the Platform deliver adequate functionality to support the BMC Youth Model? Yes/no, describe.
Implementation	What aspects of the digital health solution and its implementation have been effective within the service?

##### Online surveys and semi-structured interviews

Quantitative and qualitative data will also be collected from participants via cross-sectional online surveys using REDcap [44, 45] and semi-structured interviews will be conducted on site at participating *headspace* centres. For participants who are unable to attend a *headspace* centre, or if in-person interviews are not appropriate (i.e. due to COVID-19 health restrictions), participants will be offered telephone or online video-conference interviews using Zoom, a secure cloud-based video-conferencing service with end-to-end chat encryption. Example questions from the online surveys and semi-structured interviews are provided in Tables 2 and 3 respectively. The full version of the online survey and semi-structured interview are provided in Additional files 2 and 3: Appendices B and C, respectively.

**Table 2:** Example questions from the online survey

Topic: Views on digital health, and the adoption of the digital health solution	
Question	Answer
In the last 2 weeks, to what extent did you employ the following in your usual clinical care:	*(N/A/ not at all/ rarely/ sometimes/ very often/ always)* □ Broad, multi-dimensional assessment of needs beyond mental health, including but not limited to: physical health, daily functioning, alcohol and drug use, and social connectedness □ Outcome monitoring to routinely measure a young person’s progress using objective, standardised measures to track improvements or deterioration, for the purposes of treatment planning □ Match the ‘intensity’ of an intervention to the needs of the young person □ Shared or collaborative decision making with the young person under your care □ Etc.
What is the main reason for not always adopting any of the previous items in your usual clinical care? Please select all that apply.	□ I do not think any of the above items are important to adopt in my usual clinical care □ I am worried that the digital health solution poses a potential risk to the quality of the care provided to clients □ Time constraints □ Capacity restraints □ Etc.
When considering the positive social benefits you identified in the previous question, how much do you personally agree or disagree with the following statements?	*(Not sure/ strongly disagree/ disagree/ neutral/ agree/ strongly agree)* □ Without the Brain and Mind Centre’s digital health solution, these positive social benefits (on consumers, my standard practice or my health service) would have happened anyway □ Due to the Brain and Mind Centre’s digital health solution, other tasks I used to carry out have stopped or have been replaced □ These positive social benefits will continue in the years to come

**Table 3:** Example questions used during a semi-structured interview

Topic: Digital readiness and staff competence	
Question	Answer
When it comes to the use of digital health in your work, would you say you are keeping up, or falling behind?	□ Keeping up ▪ What helps you to keep up (e.g. personal interest, training provided by service, etc)? ▪ What are the enablers to using digital health solutions? ▪ When you describe yourself as ‘keeping up,’ to whom are you comparing yourself (e.g. colleagues, etc.)? □ Falling behind ▪ Why do you think you are falling behind? ▪ Do you prefer not to use digital health solutions in your work? ▪ Is your use of digital health solutions at work different from your colleagues? ▪ Etc.

Table 4 presents a summary of the impact evaluation outcome data collected (e.g. digital readiness and competence of staff, adoption of the digital health solution, etc), the method with which this data was collected (e.g. online survey, semi-structured interview), and the data collection timepoint.

**Table 4:** Impact evaluation outcome data collection

Data collection source	Online survey	Semi-structured interview	Implementation log	Service audit
Collection time point	Three-monthly	Three-monthly	Monthly	Three-monthly
Impact evaluation outcome collected	Adoption of digital health solution	Impact of digital health solution	Impact of digital health solution	Client safety
	Staff views on digital health	Quality, acceptability, usability of the digital health solution	Quality, acceptability, usability of the digital health solution	Client accessibility and equity
	Education and training outcomes	Education and training outcomes	Education and training outcomes	Workforce (staff numbers, FTE)
		Digital readiness and staff competence	Implementation barriers and facilitators	Service efficiency, expenditure, and cost
				Service effectiveness and outcomes
				Service continuity and coordination

Note: data collected at three-monthly timepoints (baseline, three-months, six-months, nine-months, and 12-months) will cover the preceding three months. Data collected at monthly timepoints will cover the preceding month

### Participant recruitment

#### Impact evaluation

All staff within participating *headspace* centres that are using the digital health solution will be invited to participate in the impact evaluation study (including health professionals [psychologists, psychiatrists, social workers, occupational therapists, general practitioners, nurses, etc], service managers and administrators). Additionally, relevant staff from the *headspace* centre’s lead agency and local PHN who were involved in the implementation of the digital health solution (e.g. project managers, project officers) will also be invited to participate.

Awareness of this study will be raised through the use of study advertisements (i.e. posters and postcards) displayed in staff areas, and through verbal conversations with on-site Implementation Officers one month prior to scheduled research activity to allow potential participants time to consider participation. To avoid perceived coercion, interested participants will need to contact the Implementation Officer to receive a Participant Information Sheet, which will provide further information regarding the study, and state that there is no obligation for any staff member to take part in the study, and that choosing not to participate, or withdrawing from the study, will cause no detriment to their career or future employment. Participants will be asked to complete and sign the Participant Consent Form with options for them to consent to receiving links to the baseline and follow up online surveys and semi-structured interviews, as well as to consent to having their interview scribed and audio-recorded.

### Reimbursement

If participants are likely to experience any loss of earnings (e.g. contract health professionals), then this loss may be provided for by an honorarium, in line with the Medicare rebate they would have received from the Australian public health system.

### Data analysis

Aggregate service-level outcome data will be compared across time points (baseline, three months, six months, nine months, 12 months), to identify change over time within services (specifically through calculating reliable change scores and effect sizes). As the aim of analyses is to determine whether the solution is effective for each individual service, sample size computation is not appropriate.

Data from online surveys will be analysed using descriptive statistics, as well as bivariate analyses using Fisher exact tests to evaluate group differences in each participating service. A reliability analysis will be carried out to evaluate internal consistency of the online surveys. All quantitative data will be analysed using the Statistical Package for the Social Sciences (SPSS) version 24 (IBM Corp).

Thematic analysis will be performed on qualitative data, including semi-structured interviews and implementation officer logs, to identify major themes relating to implementation of this digital solution across services, and will follow established thematic analysis techniques [46]. Interviews will be audio-recorded, transcribed and de-identified. Transcripts of both interviews and implementation officer logs will be coded using NVivo 12 software, and two independent knowledge translators from the research team will iteratively examine, discuss, and code the data, to establish a consensus regarding a coding framework.

### Data storage and security

For the duration of the research, all electronic data from service audits, Implementation Officer logs, online surveys and semi-structured interviews will be fed into a secure password-protected virtual machine within the Research Data Store provided by The University of Sydney. All service audit, Implementation Officer log, and online survey data will be non-identifiable, and semi-structured interviews will be de-identified if required. Upon completion of this research, all electronic data will be stored on a secured password-protected virtual machine within the Research Data Store provided by The University of Sydney.

### Ethics and dissemination

Ethics approval has been granted via Sydney Local Health District’s Human Research Ethics Committee (HREC) (Protocol No X18–0499 & HREC/18/RPAH/715), and via The University of Sydney HREC (Project No 2018/849). Site-specific approvals were obtained when required.

## Discussion

At the time of publication, six *headspace* centres had been recruited to this study and had commenced implementation and impact evaluation. The first results are expected to be submitted for publication in 2021.

Nationally, youth mental health is a priority as most mental disorders emerge before a young person turns 25 years old; and if not treated early enough, these disorders become lifelong impairments. Given recent world events such as the COVID-19 pandemic, there has never been a more critical time to focus on improving the mental health and wellbeing of young Australians. As a consequence, digital health models of care are now seen as crucial solutions for youth mental health services to rapidly increase access to quality mental health care [47].

The aim of this study is to describe a research protocol to evaluate the impact of implementing a digital health solution (delivered via the YMH and Technology Program) into *headspace* centres within urban and regional areas of Australia. It focuses on the impact of the digital health solution on staff and service, to better understand the importance of digital readiness, clinical competency and confidence, and the quality, usability, acceptability and overall impact of the digital health solution. The focus on staff and service-level data collection is integral to effective implementation of this digital health solution, and will provide critical feedback regarding the fit of the solution and identify service needs to ensure future successful implementations.

A consideration regarding future implementations is an evaluation of the effectiveness of an associated education and training program [36] that aims to upskill health professionals in the application of the digital health solution. Considering that clinical decisions made by health professionals are predominantly influenced by their knowledge, skills, and beliefs [13], the education and training of health professionals is central to adopting, sustaining and optimising the digital health solution in standard clinical practice.

## Supplementary Information

**Additional file 1:.** Implementation Officer Log

**Additional file 2:.** Baseline Online Survey

**Additional file 3:.** Baseline Interview Questions

## Data Availability

The dataset of this study will be made available upon reasonable request of the corresponding author.

## Abbreviations

BMC: Brain and Mind Centre
BMC Youth Model: Brain and Mind Centre Youth Model
HIT: Health Information Technology
PHN: Primary Health Network
YMH and Technology Program: Youth Mental Health and Technology Program
HREC: Human Research Ethics Committee
FGG: Future Generations Global
