# Supplementary material for: Implementing a digital health model of care in Australian youth mental health services: protocol for impact evaluation
Source: BMC Health Serv Res. 2021 May 12;21:452. doi: 10.1186/s12913-021-06394-4 (PMC8113792; doi:10.1186/s12913-021-06394-4)
Supplement: Supplementary file 1 — Additional file 1:. Implementation Officer Log [file 12913_2021_6394_MOESM1_ESM.docx]

| **IMPLEMENTATION OFFICER LOG** | | |
| --- | --- | --- |
| *Date:* |  | |
| *Service Partner(s):* |  | |
|  | *Over the last four weeks…* | |
| 1. **Service-level impacts**     1. Is the digital health solution changing and/or improving the following aspects of the mental health service? If yes, how? If no, why not?       - - 1. Clinical safety *(E.g. can risk be identified earlier? Has the digital health solution resulted in any safer outcomes for clients/patients?)*           2. Accessibility *(E.g. can clients/patients more easily access the appropriate care they need? If so, how?)*           3. Efficiency *(E.g. are there processes within the service that run quicker/smoother due to the digital health solution?)*           4. Effectiveness *(E.g. Has the digital health solution improved the effectiveness of assessment/treatment/outcomes for clients/patients?)*           5. Continuity of care *(E.g. has the digital health solution allowed improved care long-term due to data tracking?)*           6. Care coordination *(E.g. has the digital health solution allowed clients/patients to be more easily referred to the appropriate clinician/service/treatment for continued care?)*           7. The service pathway *(E.g. has the digital health solution required any changes to how the client/patient moves through the service?)*           8. The delivery of staged care *(E.g. has the digital health solution improved clinicians’ uptake of/ease of use of staged care?)* 2. Describe how the service is coping with the above changes as a result of the digital health solution and its implementation? | 2, | |
| 1. **Capacity/ readiness:**    1. Have there been any changes to the service’s capacity (e.g. client load, skills, motivation)? Yes/No describe    2. Have there been changes to the technology available to the service? (e.g. has the service been provided with iPads, laptops etc.) Yes/No, describe | a.  b. | |
| 1. **Quality and usability of the digital health solution** 2. Does the platform require modifications to improve its performance? Yes/No, describe. 3. Does the Platform deliver adequate functionality to support the BMC Youth Model? Yes/no, describe. 4. Has the service staff (clinical or administrative) provided feedback on the following:    - - - 1. The quality of the digital health solution? Yes/no, describe.          2. The usability of the digital health solution? Yes/no, describe. | a.  b.  c. | |
| 1. **Implementation:**     1. What aspects of the digital health solution and its implementation have been effective within the service?    2. What aspects of the digital health solution and its implementation have raised concerns, questions, or resistance?    3. Are there any barriers to the use of the digital health solution and its implementation? Yes/No Describe    4. If Yes, what steps (if any) have been taken to address these concerns/ barriers? | a.  b.  c.  d. | |
|  |  | |
| 1. **Staff education and training** (pertains to service staff):    1. Has training been delivered to support initial and/or ongoing use of the digital health solution and its implementation within the service? If yes, describe training provided.    2. Have additional education and training needs have been identified? If yes, describe.    3. If yes, what steps have been taken to provide additional training?    4. Have previously identified training needs been addressed? Yes/No, describe.    5. Have there been any frequently asked questions by service staff? If yes, describe. |  | |
| 1. **Other Implementation Officer observations/comments** |  | |
| 1. **SERVICE PATHWAY ASSESSMENT** | *Over the last two weeks, have there been any changes to…  [Yes/No, Describe]* | *Describe the impact(s) of the implementation of the digital health solution to this pathway.* |
| 1. Referral pathways? | 1. | 1. |
| 1. Intake/ assessment pathways? | 2. | 2. |
| 1. Treatment planning pathways? | 3. | 3. |
| 1. Intervention pathways? | 4. | 4. |
| 1. Review pathways? | 5. | 5. |
| 6. Exit service pathways? | 6. | 6. |
| 1. **STAFF PROFILE**     1. Over the last two weeks, have there been any changes to the staff profile of the service? (e.g. a change of staff roles, or staff leaving the service)  *[Yes/No]*    2. If yes, describe the impact(s) (if any) of this change on the digital health solution. | **8.**  a.  b. | |
