# Supplementary material for: Implementing a digital health model of care in Australian youth mental health services: protocol for impact evaluation
Source: BMC Health Serv Res. 2021 May 12;21:452. doi: 10.1186/s12913-021-06394-4 (PMC8113792; doi:10.1186/s12913-021-06394-4)
Supplement: Supplementary file 2 — Additional file 2:. Baseline Online Survey [file 12913_2021_6394_MOESM2_ESM.docx]

**Health Services Online Survey (Baseline)**

1. What is your gender?
   - Male
   - Female
   - Do not identify has male or female
   - Prefer not to answer
2. Are you of Aboriginal or Torres Strait Islander origin?
   - No
   - Aboriginal
   - Torres Strait Islander
   - Both Aboriginal and Torres Strait Islander
   - Prefer not to answer
3. What health service(s) are you associated with? Please select all that apply.
   - headspace Early Intervention Team
   - headspace Camperdown
   - Mind Plasticity
   - Uspace (St Vincent’s Private Hospital Sydney)

Other

1. What is your current role/discipline at your health services?
   - Aboriginal and Torres Strait Islander Mental Health Worker
   - Alcohol and Substance Use Clinician
   - Case Manager
   - Clinical Psychologist
   - Counsellor
   - Crisis Counsellor
   - Dentist
   - Dietitian
   - Family Counsellor/ Therapist
   - General practitioner
   - General psychologist
   - Intake Clinician
   - Mental health nurse
   - Neuropsychologist
   - Nurse
   - Occupational therapist
   - Occupational therapy trainee
   - Peer Support Worker
   - Psychiatrist
   - Psychiatry registrar
   - Provisional psychologist
   - Service Manager
   - Service Administrator
   - Social worker
   - Social worker trainee
   - Youth Access Clinician
   - Other (please specify)
2. How many years in total have you been practicing in this discipline/role?

*Note: please report years of experience inclusive of current and former jobs.*

- - Please input

1. Agree or disagree: I see the benefit of adopting the Brain and Mind Centre’s digital health solution as part of my work.
   *Please note: the Brain and Mind Centre’s digital health solution refers to the InnoWell Platform, supported by education and training program on the BMC Youth Model.*
   - Strongly disagree
   - Disagree to some extent
   - Neither agree nor disagree
   - Agree to some extent
   - Strongly agree
2. Do you provide care to young people accessing your health service?

- No 🡪 Skip to question Q10

Yes 🡪 continue to Q8

1. In the last 2 weeks, to what extent did you employ the following in your usual clinical care:
   *Note, please indicate if this activity was supported by your use of the InnoWell Platform.*

|  | **N/A** (not applicable to my service)  (0) | **Not at all** (with no consumers)   (1) | **Rarely**  (with a small proportion of consumers) (2) | **Sometimes** (with about half of consumers)  (3) | **Very often** (with most consumers)   (4) | **Always** (all of the time, for all consumers)  (5) | **Supported by use of the InnoWell Platform** |
| --- | --- | --- | --- | --- | --- | --- | --- |
| Broad, multi-dimensional assessment of needs beyond mental health, including but not limited to: physical health, daily functioning, alcohol and drug use, and social connectedness | ☐ | ☐ | ☐ | ☐ | ☐ | ☐ | ☐ |
| Outcome monitoring to routinely measure a young person’s progress using objective, standardised measures to track improvements or deterioration, for the purposes of treatment planning | ☐ | ☐ | ☐ | ☐ | ☐ | ☐ | ☐ |
| Assessment of clinical stage, or the practice of matching a young person’s stage of illness (e.g. stage 1a, 1b, 2+) to an appropriate level of treatment, reserving more intensive treatment to more severe clinical stages | ☐ | ☐ | ☐ | ☐ | ☐ | ☐ | ☐ |
| Match the ‘intensity’ of an intervention to the needs of the young person | ☐ | ☐ | ☐ | ☐ | ☐ | ☐ | ☐ |
| Change the ‘intensity’ of an intervention to the needs of the young person over the course of care | ☐ | ☐ | ☐ | ☐ | ☐ | ☐ | ☐ |
| Shared or collaborative decision making with the young person under your care | ☐ | ☐ | ☐ | ☐ | ☐ | ☐ | ☐ |
| The engagement of other clinicians and/or service providers in the same service to coordinate appropriate care to address the young person’s needs | ☐ | ☐ | ☐ | ☐ | ☐ | ☐ | ☐ |
| The engagement of other clinicians and/or service providers not in the same service to coordinate appropriate care to address the young person’s needs | ☐ | ☐ | ☐ | ☐ | ☐ | ☐ | ☐ |
| Assess and proactively respond to suicidal thoughts and behaviours | ☐ | ☐ | ☐ | ☐ | ☐ | ☐ | ☐ |
| Encouragement for the young person to proactively work on their mental health care through online apps and/or e-tools | ☐ | ☐ | ☐ | ☐ | ☐ | ☐ | ☐ |

1. What is the main reason for not always adopting any of the previous items in your usual clinical care? Please select all that apply.
   - I do not think any of the above items are important to adopt in my usual clinical care
   - I am worried that the digital health solution poses a potential risk to the quality of the care provided to clients
   - Time constraints
   - Capacity restraints
   - Technological limitations in my practice/ service
   - Lack of education and training
   - The technology offered does not seem advanced to enable the clinical concepts
   - Other: _________________________________

| 10. This section explores how well you feel equipped to adopt the Brain and Mind Centre’s digital health solution – specifically, the InnoWell Platform, supported by education and training on the BMC Youth Model. | **Not sure** | **Strongly disagree** | **Disagree** | **Neutral** | **Agree** | **Strongly agree** |
| --- | --- | --- | --- | --- | --- | --- |
| Sufficient training was provided to my service by a researcher(s) from the Brain and Mind Centre. |  |  |  |  |  |  |
| There was a well-coordinated effort to get clients (e.g. young people) trained by a researcher(s) from the Brain and Mind Centre in the use of the InnoWell Platform. |  |  |  |  |  |  |
| There was a well-coordinated effort to get clients (e.g. young people) trained by the service in the use of the InnoWell Platform. |  |  |  |  |  |  |
| Colleagues in my service are receptive to changes in clinical processes. |  |  |  |  |  |  |
| My service is ready to implement the Brain and Mind Centre’s digital health solution. |  |  |  |  |  |  |
| There is willingness within the service to implement the digital health solution for its intended purposes. |  |  |  |  |  |  |
| I feel confident in providing holistic assessment for my clients. |  |  |  |  |  |  |
| I feel confident in providing the best treatment matched to client needs. |  |  |  |  |  |  |
| My service already provides the best mental health care for clients. |  |  |  |  |  |  |
| The proposed digital health solution will improve care for my clients (e.g. young people). |  |  |  |  |  |  |

1. This section presents a number of statements describing recommendations from the Productivity Commission Report into mental health (2020). How closely do the following statements describe the current situation at your service?

|  | **Very inaccurate description** | **Reasonably inaccurate description** | **Accurate in some ways, inaccurate in others** | **Reasonably accurate description** | **Very accurate description** | **Don’t know or unable to decide** |
| --- | --- | --- | --- | --- | --- | --- |
| Consumers have access to technology that links to biometric monitoring (e.g. Fitbit, Healthkit, etc.) for real-time tracking of information related to their health and wellbeing. |  |  |  |  |  |  |
| Technology supports training and supervision opportunities for health professionals based in rural, regional and/or remote Australia. |  |  |  |  |  |  |
| Technology enhances assessment and referrals, and increases access to, and the range of, mental health treatments and supports. |  |  |  |  |  |  |
| Trusted technology provides safe clinical support on mental health care. |  |  |  |  |  |  |
| Technology promises that multiple gateways are available for people to seek mental health care; ultimately enabling people to access services and supports that are accessible, effective, and affordable, and match their needs and circumstances. |  |  |  |  |  |  |
| Mental health-related digital platforms currently available on the market are low-cost and accessible. |  |  |  |  |  |  |
| Mental health-related digital platforms currently available on the market are evidence-based. |  |  |  |  |  |  |
| Mental health-related digital platforms currently available on the market have minimal impact to mental health services. |  |  |  |  |  |  |
| Technology links primary and specialist care services to support clients experiencing mental ill health. |  |  |  |  |  |  |
| Technology links health professionals across different disciplines (e.g. General Practitioners with Psychiatrists/ Psychologists), ensuring that people are receiving rigorous assessment and treatment recommendations, matched to their needs. |  |  |  |  |  |  |
| Digital mental health platforms currently available on the market are offering person-centred pathways to assessment and treatment, supporting both individuals and health professionals to make decisions about the right treatment options. |  |  |  |  |  |  |
| Technology is used to support better continuity of care for those with mental ill-health; ensuring that seamless care is offered to individuals in need. |  |  |  |  |  |  |
| Education and training is widely available about the clinical use of technology for mental health care |  |  |  |  |  |  |
| Technology enhances the effectiveness of existing local mental health services |  |  |  |  |  |  |
| The mental health sector is making the best use of technology for mental health care |  |  |  |  |  |  |

1. When considering the positive social benefits you identified in the previous question, how much do you personally agree or disagree with the following statements?

|  | **Not sure** | **Strongly disagree** | **Disagree** | **Neutral** | **Agree** | **Strongly agree** |
| --- | --- | --- | --- | --- | --- | --- |
| Without the Brain and Mind Centre’s digital health solution, these positive social benefits (on consumers, my standard practice or my health service) would have happened anyway | ☐ | ☐ | ☐ | ☐ | ☐ | ☐ |
| Due to the Brain and Mind Centre’s digital health solution*,* other tasks I used to carry out have stopped or have been replaced | ☐ | ☐ | ☐ | ☐ | ☐ | ☐ |
| These positive social benefits will continue in the years to come | ☐ | ☐ | ☐ | ☐ | ☐ | ☐ |
| These positive social benefits will continue in my health service for… | Not sure | Six month or less | Between 6 months and 1 year | Between 1 and 2 years | Between 2 and 3 years | More than 3 years |
