# Supplementary material for: Implementing a digital health model of care in Australian youth mental health services: protocol for impact evaluation
Source: BMC Health Serv Res. 2021 May 12;21:452. doi: 10.1186/s12913-021-06394-4 (PMC8113792; doi:10.1186/s12913-021-06394-4)
Supplement: Supplementary file 3 — Additional file 3:. Baseline Interview Questions [file 12913_2021_6394_MOESM3_ESM.docx]

Introduction: *Thank you for taking the time to meet with me today. Together, we will discuss digital readiness and competence, and the impact and value of the digital health solution for your service. The discussion will take approximately 90 minutes, with breaks provided as needed.*

*Before we begin, I would like to ask you to complete a brief online survey asking a few questions about you, your role, any notable changes or disruptions that might have occurred at the service, and the implementation of the digital health solution into the service.*

1. What is your gender?
   - Male
   - Female
   - Do not identify has male or female
   - Prefer not to answer
2. Are you of Aboriginal or Torres Strait Islander origin?
   - No
   - Aboriginal
   - Torres Strait Islander
   - Both Aboriginal and Torres Strait Islander
   - Prefer not to answer
3. What health service(s) are you associated with? Please select all that apply.
   - headspace Early Intervention Team
   - headspace Camperdown
   - Mind Plasticity
   - Uspace (St Vincent’s Private Hospital Sydney)
   - Other
4. What is your current role/discipline at your service?
   - Aboriginal and Torres Strait Islander Mental Health Worker
   - Alcohol and Substance Use Clinician
   - Case Manager
   - Clinical Psychologist
   - Counsellor
   - Crisis Counsellor
   - Dentist
   - Dietitian
   - Family Counsellor/ Therapist
   - General practitioner
   - General psychologist
   - Intake Clinician
   - Mental health nurse
   - Neuropsychologist
   - Nurse
   - Occupational therapist
   - Occupational therapy trainee
   - Peer Support Worker
   - Psychiatrist
   - Psychiatry registrar
   - Provisional psychologist
   - Service Manager
   - Service Administrator
   - Social worker
   - Social worker trainee
   - Youth Access Clinician
   - Other (please specify)
5. How many years in total have you been practicing in this discipline/role?

*Note: please report years of experience inclusive of current and former jobs.*

- - Please input

*Thank you for that information. Do you have any questions before we begin the interview?*

**Digital readiness and competence**

*To begin, let’s talk about your use of digital health/technology. In this section, we hope to better understand how and why you and/or your service are (or are not) prepared to adapt to the use of digital technologies in mental health care.*

1. Present the Innovation Adoption Model and ask the participant:
   - How would you characterise your likelihood to adopt process changes within your service?
   - How would you characterise your likelihood to adopt new technologies within your own health care practice?
   - How would you characterise your clients’ likelihood to adopt new technologies in their mental health care?
   - How would you characterise the likelihood of your service to adopt new technologies in practice for better mental health care?


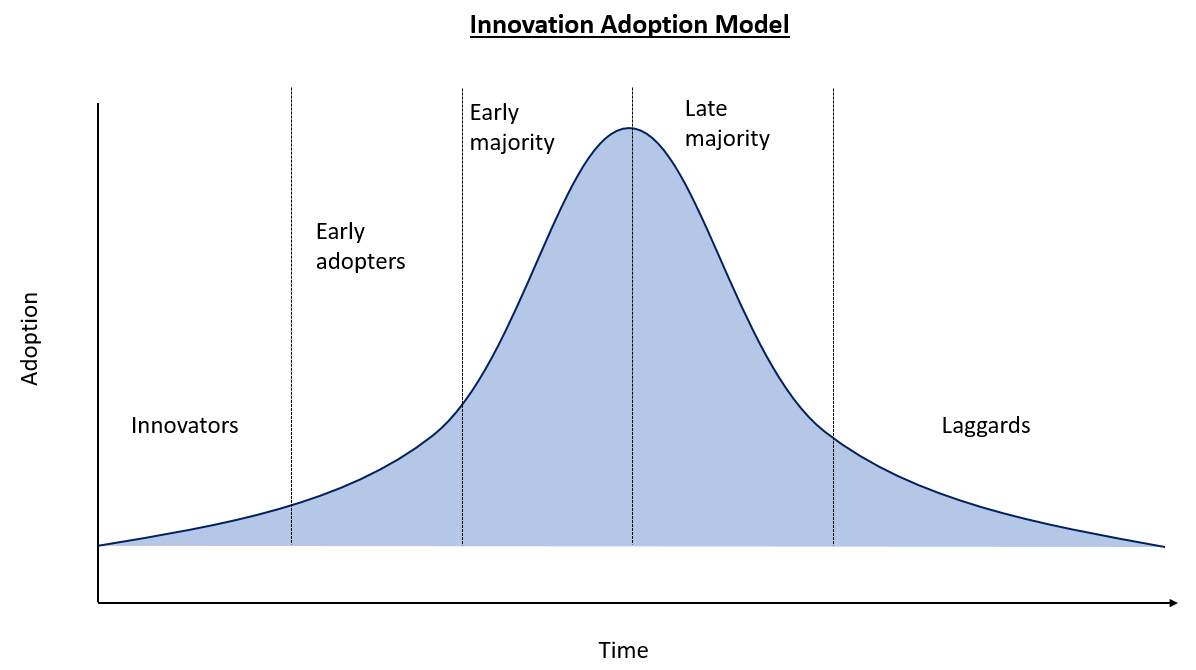


1. When it comes to digital health/technology in your work, would you say you are keeping up, or falling behind?
   - Keeping up
     - *What helps you to keep up (e.g. personal interest, training provided by service, etc.)? What are the enablers to using technology and discovering new technologies? When you describe yourself as ‘keeping up,’ to whom are you comparing yourself (e.g. colleagues, friends, family, etc.)?*
   - Falling behind
     - *Why do you think you are falling behind? Do you prefer not to use technology in your work? Is your use of technology at work different from your colleagues? Are there barriers to using technology in your work (e.g. cost, client disinterest, hardware limitations, etc.)? Is there anything that might help you to use technologies at work (e.g. time, training, financial support, improved hardware)?*
2. When it comes to digital health/technology in the workplace, is your service keeping up, or falling behind?
   - Keeping up
     - *What helps the service keep up (e.g. investment in technology, internal research and development work, support from organisational leadership, education and training, etc.)?*
   - Falling behind
     - *Why do you think the service is falling behind? Are there barriers to using technology in the service (e.g. client disinterest, hardware limitations, etc.)? Is there anything that might facilitate the use of technology in the service (e.g. training, improved hardware)?*
3. When it comes to using digital health/technology as part of their mental health care, on average are your clients keeping up or falling behind?
   - Keeping up
     - *What helps your clients to keep up (e.g. personal interest, peer support, financial resources, etc.)? What are the enablers for them to use technology and discover new technologies? When you describe your clients as ‘keeping up,’ to whom are you comparing them (e.g. other clients, colleagues, friends, family, etc.)?*
   - Falling behind
     - *Why do you think your clients are falling behind? Are there barriers for your clients to use technology in their care (e.g. lack of knowledge of available technology, lack of interest, financial limitations, etc.)? Is there anything that might help your clients to use technology in their care (e.g. training, improved access to hardware/ devices, improved access to the Internet and/ or data)?*
4. Do you try out new digital health solutions/technologies in your work?
   - *If yes, how frequently? How do you learn about new technologies (e.g. colleagues, clients, your own research)? What makes you want to use technology (e.g. to help a client, colleague recommendation, etc.)?*
   - *If no, why not? Are you skeptical of new technologies? Do you struggle to learn how to use new technologies? Is there anything that might help you to use new technologies (e.g. time, training, financial support, improved hardware)?*
5. How do you typically connect to the Internet at work?
   - *Is this different from your preferred method of connecting to the Internet outside of work (e.g., wifi, broadband, etc.)?*

**Use of the digital health solution**

*Now, tell me what you think about the implementation of the digital health solution (the InnoWell Platform) in your service.*

1. What do you anticipate the impact to be on your practice (e.gincreased clinical confidence, increased collaboration with clients regarding treatment planning, increased engagement in care from clients, changes to efficiency of care, increase case load)?
   - *Why do you think this change will happen? How do you think this will happen?*
2. What do you anticipate the impact to be on your service (e.g. more skilled staff, improved team decision making, reduced wait times, improved access)?
   - *Why do you think this change will happen? How do you think this will happen?*
3. What do you anticipate the impact to be on your clients (e.g. improved accessibility, improved ability to self-manage care, increased engagement in care from clients)?
   - *Why do you think this change will happen? How do you think this will happen?*
4. Do you think the impact will be positive? Negative? Mixed?
   - *Why?*

**Education and Training**

- Are there any education and training activities that you would like to see offered on the digital health solution?
  - *If yes, what activities? Why do you think this would be helpful? For whom do you think this would be helpful (e.g., you, your clients, your service)?*
  - *If no, why not? Are there other resources you prefer to use for education and training (e.g. professional organisation, peer supervision, etc.)? If yes, what are they and why do you prefer them over what is being offered regarding the digital health solution?*
